# A household-based survey of the morbidity profile of under-five children in Port Harcourt Metropolis, Southern Nigeria

**DOI:** 10.11604/pamj.2022.42.182.32767

**Published:** 2022-07-06

**Authors:** Tondor Cleopatra Uzosike, Ishmael Daniel Jaja

**Affiliations:** 1 Department of Community Medicine, Rivers State University, Nkpolu, Port Harcourt, Rivers State, Nigeria

**Keywords:** Morbidity profile, under-five children, household survey, Port Harcourt, Nigeria

## Abstract

**Introduction:**

the commonest causes of childhood morbidity and deaths are avoidable and curable, and have been well reported in country-wide surveys. This study was conducted to validate the locality-specific childhood morbidity profile among households in urban Port Harcourt Metropolis in the oil-rich South-South Nigeria.

**Methods:**

an observational, cross-sectional survey was conducted involving 806 mother/under-five children pairs that were randomly selected by a modified cluster sampling design. Data were collected using a pre-tested, interviewer-administered structured questionnaire that was adapted from the Nigeria Demographic and Health Survey instrument (2018), which explored information on common causes of morbidity by verbal autopsies. Analysis was done with the Statistical Package for Social Sciences, version 21.

**Results:**

the under-five children were 406 males (50.4%) and 400 females (49.6%) with an average age of 27.7 ± 17.9 months. Fever was the most frequent morbidity, reported in 364 (45.2%, 95% CI = 0.4175-0.4861) of the children, followed by cough in 362 (44.9%, 95%CI = 0.4150-0.4836), fast breathing in 49 (6.1%, 95%CI = 0.0458-0.0789), and diarrhoea in 17 (2.1%, 95%CI = 0.0139-0.0308). Symptoms of fever (chi-square = 31.117, P-value=0.001) and cough (chi-square=21.416, P-value = 0.001), were higher in the 48-59 month age group.

**Conclusion:**

febrile illness, acute respiratory tract infection, and diarrhoea disease were found to be common in under-five children in the metropolitan city of Port Harcourt. Tailored community-based health interventions and surveillance are needed to reduce the frequency of childhood morbidity and prevent mortality in this age group.

## Introduction

Child morbidity, one of the key indicators of child health, occurs as a result of preventable childhood illnesses such as malaria, acute respiratory infections (ARI), and diarrhoeal diseases which contribute substantially to mortality among children less than five years old [1]. Globally in 2019, approximately 5.2 million deaths were recorded to occur among under-five children, and from this, the highest mortality of 2.4 million occurred among neonates (less than 28 days old), while children between the ages of one to eleven months accounted for 1.5 million of the deaths and children between the ages of one to four years accounted for 1.3 million deaths [2]. Sadly, these deaths have occurred due to factors that are largely preventable with proper nutrition, complete immunization, safe water supply, or early diagnosis and treatment at the health facilities [2].

Despite the global reduction in deaths over the years among children less than five years old, Nigeria is still burdened with the under-five mortality indices that are far from meeting the targeted under-five mortality rate of below 25 deaths/1000 live births in the Sustainable Development Goal three (SDG 3). Nigeria is considered the highest contributor to global under-five deaths with a figure of 858,000 under-five deaths in 2019 and accounts for 13% of under-five deaths worldwide and 26% of the occurrence in the sub-Saharan African region [3]. Childhood morbidity is a significant contributor to the occurrence of these deaths. Worldwide, pneumonia, diarrhoea, and malaria were responsible for about 29% of under-five mortality in 2018 [4]. Reports show that almost 90% of all deaths among under-five children in Nigeria are caused by malaria, pneumonia, diarrhoea, and three vaccine-preventable diseases, including measles, pertussis, and meningitis, while infections and hypothermia are common morbidities responsible for the deaths of neonates [3]. According to the 2018 National demographic and health survey; malaria affects about 23% of children below the age of five years with 24% of them having fever two weeks before the survey, 3% were reported to have symptoms of acute respiratory infections two weeks preceding the survey and 13% of children below five years had diarrhoea two weeks before the survey [5]. Asides from the mortality caused by these illnesses, childhood morbidity severely limits and delays childhood development as it is a period when 40% and 80% of physical and cognitive growth takes place [6].

In the face of these challenges, Nigeria has taken great strides to reduce the contribution of these diseases to childhood mortality, by implementing strategies that are in line with programs developed by the World Health Organization and other agencies concerned with child health [7]. Though records show remarkable improvement following these interventions, evidenced by a reduction in under-five mortality rate from 193 to 132 deaths per 1,000 live births between 1990 and 2018 [5], under-five morbidity remains a major challenge in Nigeria. Studies have been done to identify the causes and reasons for the occurrence of morbidity among children below the age of five and several factors have been identified, including; poor environmental sanitation, lack of safe drinking water, poor maternal education, poverty, air pollution, etc. [7]. Furthermore, there are variations in the prevalence and occurrence of childhood illnesses in different geographical areas in the country, and this can hinder prompt interventions due to erroneous assumptions from generalized data. The 2018 Nigeria National Demographic and Health Survey reports that 8% of children in the North-East were more likely to have had ARI symptoms in the 2 weeks preceding the survey than children in the other zones (1%-2%) and the prevalence of fever varied from 9% in the South West to 35% in the North East while children aged 6-11 months and 12-23 months were 20% more likely to have had diarrhoea in the 2 weeks preceding the survey than children between ages 48-59 months (7%). In addition, the prevalence of diarrhoea was reported to be highest in Gombe state (35%), the Northeastern region, and lowest in southern states like Ogun and Bayelsa states (1% each) [5]. This study aims to obtain a community-based profile and prevalence of the morbidity occurring among children below the age of five in Port Harcourt Metropolis. The study assessed the overall morbidity status of under-five children in the area and examined the age group and sex prevalence and pattern of morbidity in Port Harcourt metropolis.

## Methods

**Study location:** this is a community-based study that was conducted in Port Harcourt metropolis, the capital of Rivers State. It is an urban area located between latitudes 04°43´N and 05°00´N and longitudes 06°45´E and 07°06´E, occupying an area of over 420 km^2^ in the Niger Delta. Port Harcourt has a projected population of about 3,171,076 as of 2021 [8], with twenty electoral wards. The metropolis is one of the big cities in Nigeria and a major industrial centre with several multinational oil companies, medium, and small-scale industries. Other inhabitants are engaged in fishing, farming, and trading. There are nursery, primary and secondary schools distributed in the metropolis, and a number of tertiary institutions which are mostly government-owned.

**Study population:** the study population included children aged 0-59 months, with information obtained from the mother or caregiver of the child after obtaining informed consent. A mother-child pair aged 15-49 years and less than five-year-old respectively, who have resided in the metropolis for at least one year were eligible to participate in the study. The youngest child was selected from households with more than one child below the age of five. The mother-child pair of the older child was excluded when more than one mother-child pair was found in one household.

**Study design:** a cross-sectional study design was carried out to obtain the morbidity profile of the under-five children.

**Sample size estimation:** the formula for calculating sample size for cross-sectional studies [9] was applied to determine the number of participants required for the study. Using the Nigeria Demographic and Health Survey (NDHS) malaria prevalence of 23% among under-five children with a 5% significance level, 95% confidence limit, allowing for a 20% non-response rate, and considering a design effect of two as recommended by the Center For Disease Control and Prevention (CDC) for community surveys [10], a sample size of 820 was obtained.

**Sampling technique:** a two-stage cluster sampling technique was employed to select 820 mother-child pairs from selected households using the random walk method given an assumption of one eligible mother-child pair per household [11]. In stage one, thirty enumeration areas from Port Harcourt metropolis were selected using probability proportional to size after listing all enumeration areas (EAs) in Port Harcourt metropolis and obtaining a calculated sampling interval. In stage two households were numbered and listed in each selected EA to obtain a sampling frame and 27 households were selected from each EA by simple random sampling using a table of random numbers.

### Study variables

**Dependent variable:** morbidity status indicated by presence or absence of fever, cough, fast breathing, or diarrhoea in the recent two weeks period preceding the survey, are the dependent variables. These are symptoms representing the occurrence of febrile illness for fever, acute respiratory infections for cough, then fast breathing, and diarrhoea. The two-week recall period was used to allow for comparability of results with the 2018 Nigerian Demographic and Health Surveys on child morbidity, as well as results from other community-based studies that used a two-week recall. Therefore, information on morbidity status occurring anytime from the preceding one to four days before data collection was included to minimize the recall bias.

**Independent variable:** age and sex of the child.

**Data collection:** a structured interviewer-administered questionnaire was used to obtain information from mothers on the presence or absence of fever, cough, fast breathing, and diarrhoea in their child.

**Data analysis:** questionnaires were screened for completeness, coded, and entered into Statistical Package for the Social Sciences (SPSS) version 21 software for analysis. Prevalence of findings on morbidity status of children was presented as proportions on a frequency table. Bivariate analysis using the Chi-square test was done to examine the association between demographic characteristics of the children and morbidity status. Statistical significance level was taken at p-value < 0.05 and confidence interval was set at 95%.

**Ethical consideration:** ethical approval was obtained from the Research and Ethics Committee of the University of Port-Harcourt Teaching Hospital (UPTH), with the approval number: UPTH/ADM/90/S.II/VOL.X/616. A written informed consent was got from mothers of the participants before data was collected. The data collected was kept confidential.

## Results

**Participants:** out of 820 participants selected, a total of 806 responded, giving a response rate of 98.2%.

**Descriptive data:** the average age of the children was 27.7 ± 17.9 months (Table 1). Overall, the 48 - 59 month age group had a greater percentage of children (24.8%) while the 36 - 47 month age group had the least percentage of children (17.5%). There were 406 (50.4%) males and 400 (49.6%) females. The birth order ranged from firstborn to sixth or more, and a greater proportion 261 (32.4%) of the children was in the first birth order, while the least proportion 16 (2%) of children was in the ≥ 6^th^ birth order.

**Table 1:** socio-demographic characteristics of children

Age (months)	Male (n, %)	Female (n, %)	n=806
0-11	77 (19.3)	73 (18.0)	150 (18.6)
12-23	93 (23.3)	76 (18.7)	169 (21.0)
24-35	73 (18.3)	73 (18.0)	146 (18.1)
36-47	69 (17.3)	72 (17.7)	141 (17.5)
48-59	88 (22.0)	112 (27.6)	200 (24.8)
**Birth order**			
1^st^	112 (28.0)	149 (36.7)	261 (32.3)
2^nd^	130 (32.5)	109 (26.8)	239 (29.7)
3^rd^	96 (24.0)	82 (20.2)	178 (22.1)
4^th^	40 (10.0)	42 (10.3)	82 (10.2)
5^th^	14 (8.0)	16 (3.9)	30 (3.7)
≥6^th^	8 (2.0)	2 (2.0)	16 (2.0)

**Outcome data:** the symptoms in Table 2 represent the three important childhood illnesses that lead to high morbidity among children below the age of five years. They are; malaria (Fever), acute respiratory illness, and diarrhoea. Fever (45.2%) was the most common symptom reported by the mothers to occur in the preceding two weeks before the study, followed by cough (44.9%) and then fast breathing (6.1%). The least occurring symptom was diarrhoea (2.1%). Child morbidity status was assessed by the occurrence of fever, cough, fast breathing, or diarrhoea.

**Table 2:** rank order of morbidity status of children

Variables	N	%	95% Confidence interval (CI)
Fever	364	45.2	0.4175 - 0.4861
Cough	362	44.9	0.4150 - 0.4836
Fast breathing	49	6.1	0.0458 - 0.0789
Diarrhoea	17	2.1	0.0139 - 0.0308

Multiple responses applied

### Main results

Table 3 summarizes the age distribution of these symptoms occurring in the past two weeks preceding data collection among children. The 48-59 month age group had the highest prevalence of fever 98 (26.9%) and cough 99 (27.3%) and the observation were statistically significant (p=0.001) while the group with the least prevalence of fever was the 0-11 month age group 38 (10.4%). Fast breathing also occurred more frequently among the 48-59 month age group 17 (34.7%), but this observation was not statistically significant (p=0.051), and the 0-11 month age group had the least prevalence of fast breathing 4 (8.2%). The 12-23 month age group had the highest prevalence of diarrhoea 8 (47.1%) in the past two weeks preceding this study, but this observation was not statistically significant (p = 0.064).

**Table 3:** age and sex distribution of morbidity among under-five children

Age	Fever		Cough		Fast Breathing		Diarrhea	
	Yes n (%)	No n (%)	Yes n (%)	No n (%)	Yes n (%)	No n (%)	Yes n (%)	No n (%)
0-11	38 (10.4)	112 (25.3)	42 (11.6)	108 (24.3)	4 (8.2)	146 (19.3)	2 (11.8)	148 (18.8)
12-23	89 (24.5)	80 (18.1)	83 (22.9)	86 (19.4)	6 (12.2)	163 (21.5)	8 (47.1)	161 (20.4)
24-35	75 (20.6)	71 (16.1)	70 (19.3)	76 (17.1)	9 (18.4)	137 (18.1)	4 (23.5)	142 (18.0)
36-47	64 (17.6)	77 (17.4)	68 (18.8)	73 (16.4)	13 (26.5)	128 (16.9)	1 (5.9)	140 (17.7)
48 -59	98 (26.9)	102 (23.1)	99 (27.3)	101 (22.7)	17 (34.7)	183 (24.2)	2 (11.8)	198 (25.1)
X2 (p)	31.117 (0.001)*		21.416 (0.001)*		9.443 (0.051)		8.887 (0.064)	
**Sex**	**Fever**		**Cough**		**Fast Breathing**		**Diarrhea**	
	**Yes n (%)**	**No n (%)**	**Yes n (%)**	**No n (%)**	**Yes n (%)**	**No n (%)**	**Yes n (%)**	**No n (%)**
0-11	177 (48.6)	229 (51.8)	189 (52.2)	217 (48.9)	23 (46.9)	383 (50.6)	7 (41.2)	399 (50.6)
12-23	187 (51.4)	213 (48.2)	173 (47.8)	227 (51.1)	26 (53.1)	374 (49.4)	10 (58.8)	390 (49.4)
X2 (p)	0.809 (0.368)		0.888 (0.346)		0.246 (0.620)		0.587 (0.443)	

X2 =Chi-square, p=p-value, *=Significant

Table 3 also shows that fever was more prevalent among females in 187 (51.4%) compared to males in 177 (48.6%) but males had a higher prevalence of cough in 189 (52.2%) compared to females in 173 (47.8%). Females had a higher occurrence of fast breathing 26 (53.1%) compared to the males 23 (46.9%), and a higher prevalence of diarrhoea 10 (58.8%) compared to the males 7 (41.2%), but the observations were not statistically significant.

## Discussion

The most frequently occurring symptom of morbidity among children was fever followed by cough, then fast breathing, and lastly, diarrhoea. The high prevalence of fever in this study is similar to observations made by Egbewale *et al*. [12], who reported fever as the most prevalent cause of morbidity among under-five children in Osun state. Sa´ad *et al*. [13] in north-western Nigeria, reported fever as the most prevalent symptom occurring among under-five children admitted into the pediatric ward, and Ezeonwu *et al*. [1] also observed a high prevalence of fever as the most common morbidity among the children admitted into the Federal Medical Centre Asaba. Findings from this study on child morbidity are also similar to the 2018 NDHS report [5] which has fever as the most common symptom occurring among under-five children two weeks preceding the survey, this was followed by diarrhoea and acute respiratory infection. Malaria may be the reason for the high prevalence of fever, as Nigeria is a malaria-endemic zone. The finding from this study also corroborates with reports from the World Health Organization, stating that children less than 5 years are more susceptible to having Malaria, especially in African countries. However, because other childhood illnesses such as gastroenteritis, sinusitis, etc. can present with symptoms of fever, this finding should cause an increase in health-seeking behaviour of caregivers to promptly diagnose the cause of fever and treat the child accordingly.

The findings of this study on child morbidity, differ from observations made by other researchers in Nigeria [14] who observed a high prevalence of pneumonia in almost fifty percent of the children, as the most common morbidity among under-five children admitted into a tertiary hospital. Our finding of fever as the most frequent cause of morbidity also differs from reports made by a researcher in India [15] who observed that acute respiratory infections and diarrhoea were the major symptoms of morbidity observed among the children in the preceding two weeks before their survey. Geographic and seasonal variations on the occurrence of diseases, climatic conditions, and socioeconomic factors may be reasons for these observed differences. When the occurrence of morbidity was assessed by age group, the prevalence of fever was significantly higher among the 48-59 month age group. This occurrence may be because this age group is part of the pre-school children who probably have a higher risk of contracting malaria and other communicable diseases from other children at the nursery schools compared to the infants in the lower age group.

There was a higher prevalence of cough among the males compared to females, but this observation was not significant. Cough was also significantly prevalent in the 48 - 59 month age group, implying a higher occurrence of acute respiratory infection in this age group, and this is also one of the common morbidities observed among children in the pre-school years. Fast breathing was also higher in the 48 - 59 month age group and although this observation was not significant, it adds to the fact that children in this age group may have more episodes of acute respiratory infections compared to other age groups. Diarrhoea was the least frequently occurring morbidity among children in this study, it may be that the mothers have good handwashing practices or there is a safe drinking water supply in the urban area of study. Other studies have documented similar observations [16] where diarrhoea was less prevalent than acute respiratory infection among children below the age of five years. The prevalence of diarrhoea in this study is however much lower than the prevalence observed by other researchers among under-five children in Nigeria, India, and Ethiopia [1,5,14,16,17]. This observed difference may be attributed to differences in study location and designs, health-seeking behaviour, educational and socio-economic status of the participants.

### Limitations

Findings may apply specifically to the area of study and cannot be generalized. Further information needed to assess the socio-economic status of care givers and morbidity status of under-five children such as clinical examinations and biomedical investigations were not done due to limited financial resources required for the calculated sample size of participants. This however provides a platform for further studies to be conducted.

## Conclusion

This study shows that symptoms of fever, cough, fast breathing, and diarrhoea still occur commonly among under-five children in Port Harcourt metropolis and these are principal symptoms of malaria, acute respiratory infections, and diarrhoea that are leading causes of childhood mortality and a significant public health concern. For practice, community health interventions such as; promoting exclusive breastfeeding, supporting continued breastfeeding and complementary feeding, providing and promoting the utilization of long-lasting insecticide-treated nets for children below the age of five years, improving health-seeking behaviour, prevention and prompt management of childhood malaria, pneumonia, and diarrhoea, promoting routine immunization for children and prompt management of acute malnutrition should be put in place to reduce the prevalence of these preventable diseases and ultimately reduce childhood mortality.

### What is known about this topic

- *The global and national prevalence of childhood illnesses and mortality are available;*
- *There is generalized information about the pattern of childhood morbidity*.

### What this study adds

- *Validates the localized prevalence and morbidity pattern of childhood illnesses in Port Harcourt Metropolis;*
- *Provides information that encourages surveillance activities and preventive health interventions to reduce the prevalence of childhood morbidity and mortality*.
